# The drosomycin multigene family: three-disulfide variants from *Drosophila takahashii* possess antibacterial activity

**DOI:** 10.1038/srep32175

**Published:** 2016-08-26

**Authors:** Bin Gao, Shunyi Zhu

**Affiliations:** 1 Group of Peptide Biology and Evolution, State Key Laboratory of Integrated Management of Pest Insects and Rodents, Institute of Zoology, Chinese Academy of Sciences, 1 Beichen West Road, Chaoyang District, Beijing 100101, China

## Abstract

Drosomycin (DRS) is a strictly antifungal peptide in *Drosophila melanogaster*, which contains four disulfide bridges (DBs) with three buried in molecular interior and one exposed on molecular surface to tie the amino- and carboxyl-termini of the molecule together (called wrapper disulfide bridge, WDB). Based on computational analysis of genomes of *Drosophila* species belonging to the Oriental lineage, we identified a new multigene family of DRS in *Drosphila takahashii* that includes a total of 11 DRS-encoding genes (termed *DtDRS-1* to *DtDRS-11*) and a pseudogene. Phylogenetic tree and synteny analyses reveal orthologous relationship between *Dt*DRSs and DRSs, indicating that orthologous genes of *DRS-1*, *DRS-2*, *DRS-3* and *DRS-6* have undergone duplication in *D. takahashii* and three amplifications (*DtDRS-9* to *DtDRS-11*) of *DRS-3* have lost WDB. Among the 11 genes, five are transcriptionally active in adult fruitflies. The ortholog of DRS (*Dt*DRS-1) shows high structural and functional similarity to DRS while two WDB-deficient members display antibacterial activity accompanying complete loss or remarkable reduction of antifungal activity. To the best of our knowledge, this is the first report on the presence of three-disulfide antibacterial DRSs in a specific *Drosophila* species, suggesting a potential role of DB loss in neofunctionalization of a protein via structural adjustment.

*Drosophila* flies live in rotten fruits and vegetables where there are a large number of filamentous fungi competing for nutrients^1^. Drosomycin (DRS) is the first inducible antifungal peptide isolated from the haemolymph of immune-challenged *Drosophila melanogaster*, which selectively destroys spores and hyphae of filamentous fungi via a partial lysis mode^2^,^3^. Structurally, DRS is composed of 44 residues with a typical cysteine-stabilized α-helical and β-sheet (CSαβ) fold that includes an α-helix and a three-stranded β-sheet^4^. DRSs from different species of *Drosophila* characterized so far contain eight cysteines (named Cys^1^ to Cys^8^) that form four intramolecular disulfide bridges (DBs) with three buried in the molecular core (Cys^2^-Cys^5^, Cys^3^-Cys^6^ and Cys^4^-Cys^7^) (named core disulfide bridge); and one exposed onto the molecular surface to link two β-strands (β1 and β3) in the molecular termini (Cys^1^-Cys^8^)^5^ (Fig. 1). This unusual DB, also occurring in structurally homologous scorpion sodium channel toxins, was firstly termed wrapper disulfide bridge (WDB) by Hammock and colleagues^6^. Four-disulfide DRSs hold a compact structure, rendering them remarkably stable and highly resistant to heat and proteases^7^. Site-directed mutagenesis studies reveal a key functional role of seven charged and one aromatic residues in DRS^3^,^8^ (Fig. 1), in which four single mutations (i.e. D^1^, R^20^, R^21^ and K^38^) resulted in complete loss of its antifungal activity.

As one target gene of the Toll signal pathway, *DRS* can be rapidly synthesized in the fat body and secreted into haemolymph up to a concentration of 100 μM in response to microbial infection^9^. *DRS* in *D. triauraria* is also up-regulated following immune challenges in diapausing adults^10^. Besides inducible systemic expression in the fat body, *DRS* is also constitutively expressed in a variety of epithelial tissues of larvae, pupae and adults, which is independent of the Toll pathway^11^. In addition to *DRS*, the *D. melanogaster* genome also encodes six additional paralogous genes (*DRS-1* to *DRS-6*), all located within a 56 kb region of the left arm of chromosome 3 with three distinct clusters (C1, C2 and C3)^12^,^13^. Similar to *DRS*, *DRS-2* is also expressed in three developmental stages of fruitflies (larvae, pupae and adults); *DRS-3* - *DRS-5* are only expressed in larvae and adults, whereas *DRS-1* and *DRS-6* were not expressed in all the developmental stages of *Drosophila*^14^. When challenged, the expression level of *DRS*, *DRS-2* and *DRS-3* were up-regulated but only *DRS* was strongly induced. *DRS-1* and *DRS-6* are not expressed even in the presence of microbes^13^. Homologous genes of *DRS* are also found in the *melanogaster* species group, all retained as a multigene family^15^,^16^.

In this work, we conducted a large scale of survey on all sequenced *Drosophila* genomes, from which we identified a complete set of DRS peptides in 14 species, including a new multigene family of DRSs (designated *Dt*DRS) in *Drosophila takahashii*, a Southeast Asian species belonging to the *takahashii* subgroup. It is striking that two members with three DBs (*Dt*DRS-11 and *Dt*DRS-11d) possess antibacterial activity accompanying complete loss or remarkable decrease of antifungal function. A combination of sequence, structural and functional analyses suggests that a DB loss-mediated structural modification is likely implicated in the emergence of antibacterial function in the two WDB-deficient DRSs.

## Results

### Gene Expansion of the DRS Family in *D. takahashii*

By TBLASTN search of the whole-genome shotgun contigs (wgs) databases of *Drosophila* in GenBank (March 18, 2015), we identified all *DRS*-type antifungal peptide genes in 14 species, which all belong to the *Drosophila melanogaster* species group^17^. Figure 2 shows their genomic position. As observed previously^13^,^16^, the genomic arrangement of the *DRS* family members is conserved in the Oriental lineage where three distinct clusters (C1 to C3) are separated by two long spacers of 18–38 kb, which is different from species from the *ananassae* and *montium* subgroups whose *DRS* gene clusters display a relatively scattered distribution pattern (Fig. 2). In view of basal position of these two subgroups within the *melanogaster* species group^18^, an ancient *DRS* could have undergone independent expansion between these basal species and the monophyletic Oriental lineage. In spite of overall conservation in the Oriental lineage, gene turnover frequently occurred in this family, which can be outlined as follows: (*a*) Seven paralogous genes are conserved among *D. elegans*, *D. erecta*, *D. simulans* and *D. melanogaster*, suggesting that they originated by early gene duplication in the common ancestor of the Oriental lineage; (*b*) *D. yakuba* and *D. sechellia* lost one member in C3, and one member in this cluster became a pseudogene in *D. biamipes*; (*c*) *D. ficusphila* contains the smallest gene number on account of loss in C2 and C3; (*d*) Both *D. rhopaloa* and *D. takahashii* have undergone gene duplication in C3, and in *D. takahashii* C2 has also expanded to four paralogous genes (Fig. 2).

**Figure 1:**
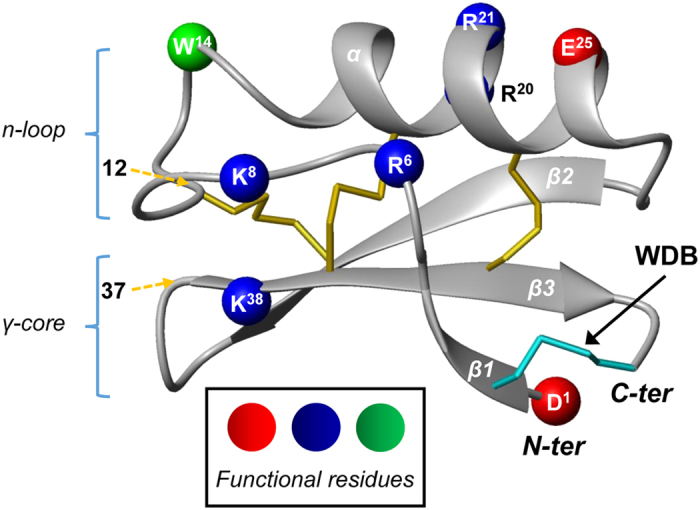
Structure of DRS. Ribbon diagram of DRS structure (PDB entry 1MNY) is displayed by MOLMOL^50^ with functional residues^7^,^8^ highlighted in color: *blue* (basic), *red* (acidic), and *green* (aromatic). Sites 12 and 37 contain three-disulfide *Dt*DRS-specific residues (M^12^ and F^37^), indicated by dotted arrows in *yellow*.

*D. takahashii* contains 11 homologous genes of *DRS* (termed *DtDRS-1* - *DtDRS-11*) and one pseudogene with a premature stop codon in the signal peptide-encoding region (named *PseudoDtDRS*) (Fig. 3). Unlike DRSs from other *Drosophila* species, the *Dt*DRS multigene family contains three members (*Dt*DRS-9 to *Dt*DRS-11) without WDB. The loss attributes to mutations of codons encoding two cysteines (Cys^1^ and Cys^8^, TGT) into a codon of Phe (TTT) and a stop codon (TGA). From the genome of *D. lutescens*, a sibling species of *D. takahashii*, we also amplified a WDB-deficient DRS (designated *Dl*DRS, Supplementary Figs S1 and S2), suggesting that the history of these unique DRS molecules could trace back to the common ancestor of the *takahashii* subgroup. *Dt*DRSs and DRSs both share 15 identical sites, including six cysteines, three glycines (Gly^5^, Gly^9^, and Gly^31^), two acidic residues (Asp^1^ and Glu^42^), two tryptophans (Trp^14^ and Trp^40^), one serine (Ser^4^), and one histidine (His^32^) (numbered according to DRS) (Fig. 3). Two of them (Asp^1^ and Trp^14^) have been identified as functionally important residues of DRS involved in the interaction with fungi^3^,^7^,^8^ (Fig. 1).

### Synteny of the DRS Clusters between *D. takahashii* and *D. melanogaster*

Our phylogenetic analysis established clear orthologous pairs between members from the DRS and *Dt*DRS multigene families: *DRS-2* and *DtDRS-4*/*PseudoDtDRS*; *DRS-3* and *DtDRS-9 - DtDRS-11*; *DRS-4* and *DtDRS-6*; *DRS-5* and *DtDRS-2*; *DRS-1* and *DtDRS-3*/*DtDRS-5*; *DRS-6* and *DtDRS-7*/*DtDRS-8*; *DRS* and *DtDRS-1* (Fig. 4A). *DRS-1*, *DRS-2*, *DRS-3* and *DRS-6* have undergone gene duplication in *D. takahashii*. Given that *DtDRS-9 - DtDRS-11* cluster together to constitute a monophyletic clade in the phylogenetic tree (Fig. 4A), it is reasonable to infer that the loss of WDB in *D. takahashii* occurred only once during evolution. Figure 4B shows the synteny relationship between *Dt*DRS and DRS genes, giving strong support for the same conclusion based on the phylogenetic analysis (Fig. 4A), in particular, for the branches with low bootstrap values (e.g. <50%).

### Molecular Characterization of DtDRS Genes

To isolate cDNA clones encoding *Dt*DRS-1 to *Dt*DRS-11, we designed a series of degenerate forward primers (Table S1) in combination with 3AP to perform RT-PCR^19^. Our RT-PCR experiments confirmed that four *Dt*DRS genes were transcriptionally active in *D. takahashii* adults without experimental stimulus, including *DtDRS-1*, *DtDRS-2*, *DtDRS-6*, and *DtDRS-11* (Fig. 5A), which correspond to their orthologs in *D. melanogaster* (*DRS*, *DRS-5*, *DRS-4*, and *DRS-3*). All these *D. melanogaster* genes are also transcriptionally active in adults in the absence of experimental infection^14^. *DtDRS-4* is a gene whose transcription depends on microbial stimulus, indicating its inducible feature. This appears to be different from its othologous gene - *DRS-2.* In adult *D. melanogaster*, *DRS-2* is transcribed in a constitutive manner (Fig. 5A). Using degenerate *Dt*DRS-3/5-F and *Dt*DRS-7/8-F primers, we failed to amplify PCR products for four genes (*DtDRS-3,* −*5,* −*7* and −*8*) from the first-strand cDNA templates prepared from both non-challenged and challenged adult fruitflies. Using degenerate *Dt*DRS-2/4/9-10-F, we obtained PCR products from the challenged or non-challenged cDNA template, but all clones sequenced carry inserts encoding *DtDRS-2* or *DtDRS-4* without *DtDRS-9* and *DtDRS-10*. Among these untranscribed genes, *DtDRS-3*/*DtDRS-5* are orthologous to *DRS-1* and *DtDRS-7*/*DtDRS8* to *DRS-6* (Fig. 4B), and interestingly these two orthologous genes in *D. melanogaster* are also transcriptionally inactive in adults and other developmental stages and even after challenge, suggesting an overall conserved transcriptional pattern between the two multigene families. However, considering only adult fruitflies analyzed for *DtDRS* genes, it is likely that these untranscribed genes are functional in other developmental stages or in response to specific microbial infections given that they are conserved over more than 10 million years. Sequence analysis of the isolated cDNA clones revealed some polymorphic sequences for the transcribed *Dt*DRSs (Fig. 5B; Supplementary Fig. S2).

### Peptide Identification

There was no precedent for a DRS with three DBs in *Drosophila* reported so far. To study the potential function of these unusual peptides, we chose *Dt*DRS-11 and *Dt*DRS-11d, a cloned polymorphic cDNA sequence of *Dt*DRS-11, as representatives for chemical synthesis. They both differ by four residues (L3K, M13A, T35S and E43M) (Fig. 6A). Oxidized *Dt*DRS-11 and *Dt*DRS-11d were produced via *in vitro* folding from their reduced peptides, with retention time of 18.5 and 22.5 min, respectively, on a C_18_ column (Fig. 6B). Their experimental molecular weights (MWs) were 4800.16 and 4859.8 Da, as determined by MALDI-TOF (Fig. 6C), matching their calculated MWs (Fig. 6A). To study the potential structural and functional effect of WDB in DRS-3, we also chemically synthesized and oxidized its WDB-deficient variant (termed DRS-3-WDB) (Fig. 6). In addition, using a prokaryotic system, we prepared recombinant *Dt*DRS-1, the ortholog of DRS, for comparison with the WDB-deficient DRSs at structural and functional levels. The reason we chose recombinant expression of *Dt*DRS-1 was because there was difficulty in the chemical synthesis of this peptide with four disulfide bridges. From the chemical nature, peptides derived from recombinant or chemical synthesis are the same so long as they are characterized by standard biochemical techniques, such as RP-HPLC, MALDI-TOF and circular dichroism (CD), as described in this work. Recombinant *Dt*DRS-1 was eluted at 22.4 min of retention time and an experimental molecular mass of 4904 Da, well matching its theoretic molecular mass of 4902 Da (Fig. 6). The eluted peptides were further purified by RP-HPLC to ensure their purity >95%.

### Functional Divergence between Three- and Four-Disulfide DRSs

To assess potential antimicrobial function of *Dt*DRS-1, *Dt*DRS-11, *Dt*DRS-11d and DRS-3-WDB, we firstly assayed their effect on a series of filamentous fungi and the yeast *Candida albicans*. As a result, we found that *Dt*DRS-1 had highly similar antifungal spectrum and potency to DRS, both inhibiting the growth of *Aspergillus fumigatus* (strain CEA17 other than YJ-407), *A. nidulans* (strains A28 and RCho15), *A. niger*, *Geotrichum candidum*, and *Neurospora crassa* with lethal concentrations (C_*L*_) ranging from 0.1–2.6 μM (Table 1). Like DRS, *Dt*DRS-1 is also a strictly antifungal peptide without activity on the bacteria tested here. The most remarkable discovery here is that *Dt*DRS-11d has lost its antifungal function but evolved activity on two Gram-positive bacteria *Bacillus megaterium* and *Micrococcus luteus* (Fig. 7A) with a C_*L*_ of 0.98–1.08 μM (Table 1). Similarly, *Dt*DRS-11 is also an antibacterial peptide but with some activity against *N. crassa* (Table 1). Different from these two naturally-occurring WDB-deficient DRSs, the engineered DRS-3-WDB exhibited antifungal activity on two species (*G. candidum* and *N. crassa*) with a C_*L*_ of 3.72–4.89 μM but no activity on the bacteria used here (Table 1).

Because many antibacterial peptides kill their targets via a membrane disruption mechanism^20^, we examined a possible impact of *Dt*DRS-11 and *Dt*DRS-11d on membrane permeability of *B. megaterium* cells via propidium iodide (PI), a fluorescent nucleic acid-binding dye. The results showed that these two peptides at 5× C_L_ caused an immediate fluorescence increase upon exposure of the peptides even though the effect is much milder than that observed with the positive control meucin-18^21^, indicating that bacterial membrane integrity was affected. On the contrary, no fluorescence increase was observed after *B. megaterium* cells were exposed to vancomycin at 10× C_L_ (Fig. 7B). To evaluate the stability of the WDB-deficient peptide *Dt*DRS-11, we assayed its antimicrobial activity in water, insect saline or insect haemolymph. In these three environments, *Dt*DRS-11 displayed similar activity (Fig. 8), revealing its resistance on insect blood proteases.

### Structural Basis of Functional Divergence

To understand the structural basis of antibacterial activity in both *Dt*DRS-11d and *Dt*DRS-11, we compared their CD spectra with those of the three antifungal DRSs, including DRS, *Dt*DRS-1 and DRS-3-WDB (Fig. 9). It is known that DRS adopts a rigid and compact structure with a high content of α-helix (25%) and β-sheet (29.5%)^22^. The CD spectra of DRS were identified by maxima at 188 nm and minima at 207 nm, indicative of the presence of a CSαβ structure^23^. In addition to these two typical signals, it had one negative band arround 217–218 nm (Fig. 9), previously seldom observed in members from the same structural superfamily, such as scorpion Na^+^ channel toxins^23^,^24^. The negative band at this position is usually ascribed to β-sheet and its presence thus reveals a high content of β-sheet residues in DRS, as mentioned above. The CD spectrum of *Dt*DRS-1 was nearly the same with that of DRS (Fig. 9A), in accordance with their functional similarity (Table 1). In comparison with DRS, the three WDB-deficient peptides displayed clearly visible modifications in their CD spectra: (*a*) In *Dt*DRS-11d and DRS-3-WDB, the negative band at 217–218 nm disappeared whereas *Dt*DRS-11 remained but the intensity slightly decreased as compared to DRS (Fig. 9B–D), indicating that these three WDB-deficient peptides had a lower content of β-sheet than DRS; (*b*) The CD spectra of *Dt*DRS-11d and *Dt*DRS-11 both crossed the baseline once at 192 nm, blue-shifted 3 nm relative to DRS (195 nm) (Fig. 9), and their negative minima were also blue-shifted from 206 nm of DRS to 203 nm of *Dt*DRS-11d and 204 nm of *Dt*DRS-11 (Fig. 9). No such shift was observed in DRS-3-WDB. In the two antibacterial variants, the shifted minima next to 202 nm, a signal for random coli, suggesting their structures were more flexible than DRS and DRS-3-WDB.

The CD spectra were analyzed by CDSSTR to estimate percentages of peptide secondary structure element contents^25^ (Table 2). For all calculations, the NRMSD values^26^ ranged from 0.015 to 0.025 (Table 2), suggesting a good correlation between them. The results showed that the thee WDB-deficient peptides had similar α-helical contents (18–21%) to DRS (20%) (Table 2), indicating that the loss or deletion of WDB led to no remarkable impact on the α-helical formation. However, such modification resulted in a significant reduction in the β-sheet content from 27% of DRS to 22% of DRS-3-WDB and 16% of *Dt*DRS-11 and *Dt*DRS-11d, in line with the disappearance or the intensity decrease of the band at 217–218 nm in their CD spectra (Fig. 9; Table 2). According to the unordered contents, we can rank the structural rigidity of these peptides as follows: DRS = *Dt*DRS-1 > DRS-3-WDB > *Dt*DRS-11 > *Dt*DRS-11d, suggesting that more structural flexibility derived from the evolutionary loss of WDB in *Dt*DRS-11 and *Dt*DRS-11d might be a direct cause of the emergence of antibacterial activity from a four-disulfide DRS scaffold. This analysis also provides a reasonable structural explanation for the lack of antibacterial activity in DRS-3-WDB.

## Discussion

### Gene Duplication and Positive Selection

Gene duplication followed by positive selection represents a major event in the evolution of immune genes, presumably due to the need to cope with rapidly diversifying pathogens. However, three classical statistic models, including M2a and M8 implemented in PAML^27^, and mechanistic-empirical model (MEC) implemented in Selecton, which takes into account the physicochemical properties of amino acids^28^, all detected no positive selection signals in the *Dt*DRS multigene family (data not shown), in agreement with several previous studies on the evolution of DRS in other *Drosophila* species^15^,^16^. It is known that the absence of positive selection is common to *Drosophila* antimicrobial peptide (AMP) gene families^29^, which might be related to two factors: (*a*) non-coevolving saprophytic organisms *Drosophila* meet, and (*b*) multiple AMP genes induced by infection, both leading to selection for speed and efficiency of expression of AMPs towards infection rather than amino acid modification via accelerated evolution^16^. From a functional viewpoint, the absence of adaptive amino acid substitutions in these two multigene families (DRS and *Dt*DRS) is also likely due to the constraint of their potential house-keeping functions beyond immunity in development, diapause, fertility and lifespan^30^,^31^. Also, the power limitation of statistical approaches is another reason of detecting no positive selection because our experimental data have clearly demonstrated that cysteine mutations-associated functional diversification had occurred between three- and four-disulfide-bridged members of the DRS family.

### Contribution of Gene Duplication to *D. takahashii*

Several lines of evidences suggest that although *D. takahashii* has more DRS genes by duplication, its DRS-based antifungal immunity could be similar to *D. melanogaster*: (*a*) Firstly, DRS is an important component of antifungal defense in *D. melanogaster*^32^. The ortholog of DRS in *D. takahashii* (*Dt*DRS-1) possesses nearly the same potency against filamentous fungi (Table 1); (*b*) Secondly, *Dt*DRSs and DRSs exhibit a similar transcriptional pattern, both having five transcriptionally active orthologs in adult fruitflies; (*c*) Thirdly, genes derived from the C2 cluster all are transcriptionally inactive in our study. However, some members of the *Dt*DRS family conferring antibacterial immunity are not still reported in *D. melanogaster*. It remains an open question whether these species-specific duplicates contributes to other biological processes, as mentioned above^31^. Given that gene duplicates tend to have divergent expression patterns^33^, a detailed comparison of these differentials between the *DRS* and *Dt*DRS families will help understand the biological and evolutionary significance of gene duplication in *D. takahashii*.

### DB Loss and Functional Neofunctionalization

**Figure 2:**
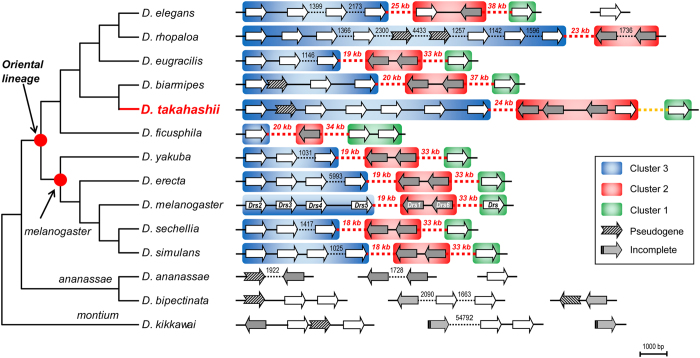
The *DRS* cluster in the *Drosophila melanogaster* species group. The phylogeny (left) is constructed based on nucleotide sequences of *RP49*. Red circles represent nodes of monophyletic Oriental lineage and the *melanogaster* subgroup. *DRS* genes are represented by arrows and different fillcolors (white and grey) refer to the orientation of genes; diagonal arrows indicate pseudogenes and virtual end arrows indicate incomplete sequences. Clusters 1–3 of the DRS multigene family are shadowed in color. Nucleotide lengths are shown in bp (short) or kb (long) when they are not scaled up.

As mentioned in Introduction, WDB is one highly exposed disulfide bridge related to peptide function. Deleting the WDB of the scorpion Na^+^ channel toxin BmKM1 dramatically reduced its potency due to destruction of a local functional region stabilized by this WDB^34^. Evidence in favor of functional importance of WDB in the DRS family members include: (*a*) All the four-disulfide DRS homologs characterized so far (e.g. DRS-2 and *Dt*DRS-1) exhibit strictly antifungal activity with a rigid structure^14^; (*b*) Functional exertion of DRS depends on a rigid scaffold stabilized by the WDB to sustain its scattered functional sites onto the molecular surface (Fig. 1). This is consistent with the absence of antifungal activity in *Dt*DRS-11d and the weak antifungal activity in *Dt*DRS-11 even if they both possess nearly identical functional amino acids to DRS (Figs 1 and 2). The increase in the unordered content accompanying the decrease in the β-sheet content in both *Dt*DRS-11d and *Dt*DRS-11 could attribute to the N-terminal rigid structure destroyed due to the WDB loss and thus a flexible N-terminus renders the functional Asp1 in a position unsuitable for interaction with fungi^7^,^8^ (see Fig. 1). On the contrary, a conformationally flexible structural region is functionally important in peptide’s binding to bacterial membrane. For example, the long N-terminal loop is a key functional region of insect defensins in bacterial killing^35^ and a series of mutational experiments have shown that a well-defined CSαβ-type defensin structure is not an advantage in its antibacterial function^36^. This reasonably explains why a structurally more rigid four-disulfide DRS lacks antibacterial activity while a structurally looser three-disulfide DRS possesses such activity. Apart from the cysteine loss leading to structural and functional changes described here, reduction of DBs has also been found to unmask potent antimicrobial activity of human β-defensin-1^37^. In addition, recent studies demonstrated that DBs in several cysteine-rich antibacterial peptides (e.g. human β-defensin-3, the designed *Nv*BH, and porcine PG-1) are dispensable for their function^38^,^39^,^40^. Taken together, all these observations support a role of the WDB loss in developing antibacterial activity from a rigid scaffold. The membrane-disruptive activity of *Dt*DRS-11d and *Dt*DRS-11 (Fig. 7B) suggests their ability in forming an amphiphilic architecture in a membrane environment via structural flexibility^21^.

**Figure 3:**
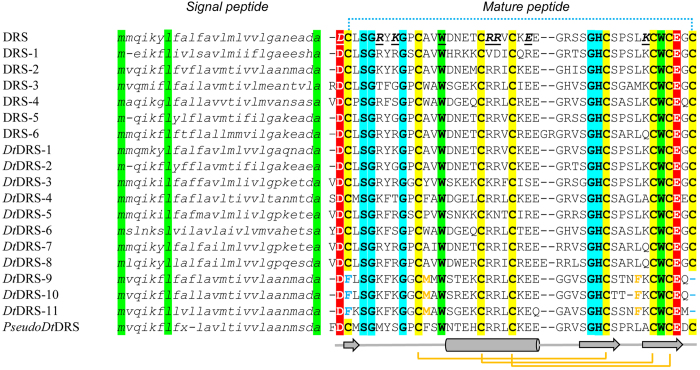
Multiple sequence alignment (MSA) of the precursor amino acid sequences of *Dt*DRSs and DRSs. Hydrophobic or aromatic residues are shadowed in green, hydrophilic in cyan, acidic in red, and cysteines in yellow. Secondary structure elements (cylinder: α-helix; arrow: β-strand) and disulfide bridge connectivities are extracted from the structural coordinates of DRS with WDB represented by a dotted line. “x” in *pseudoDt*DRS indicates position of one nucleotide deletion resulting in a premature stop codon.

**Figure 4:**
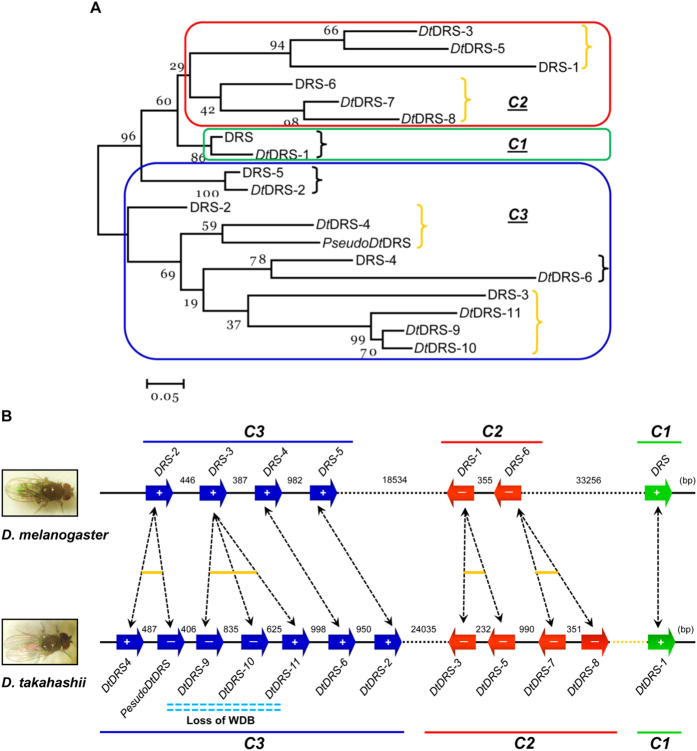
Evolutionary relationship between *Dt*DRSs and DRSs. **(A)** A neighbor-joint (NJ) tree reconstructed by MEGA5^49^. Branches showing orthologous relationship are indicated by brace and those with species-specific gene duplication in orange. Rounded rectangles mark three clusters of the DRS family. Bootstrap support values as percentage (1000 replications) are shown on each branch point of the tree. Scale bar indicates 0.05 amino acid substitutions per site. (**B)** Genomic arrangement and synteny of the *DRS* cluster in *D. melanogaster* and *D. takahashii*. Arrows in blue and red refer to orientation of genes and the sign, + or −, represents transcription or no transcription when identified by RT-PCR. Dotted arrows indicate orthologous relationship between *Dt*DRSs and DRSs, and gene duplication in *D. takahashii* is indicated in yellow. Double dotted lines in light blue indicate members with three DBs.

**Figure 5:**
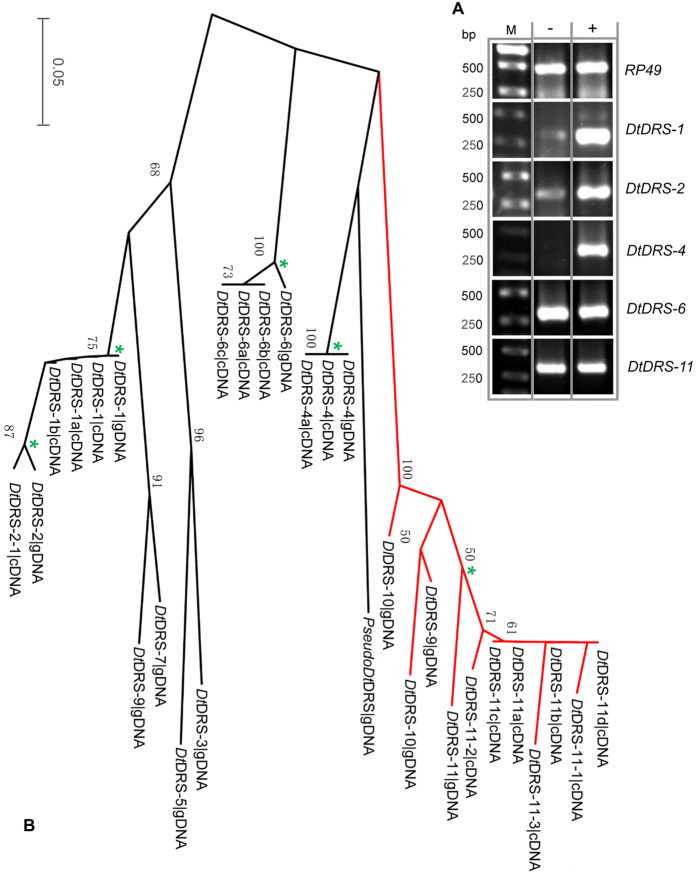
cDNA cloning of *Dt*DRSs. **(A)** RT-PCR of *DtDRS* cDNAs. M, DNA marker. −: non-challenged; +: challenged. The microbes used in the challenge were *M*. *luteus* and *N. crassa*. *RP49* was used as control; (**B)** An NJ tree based on deduced protein sequences inferring the correspondence between genomic (gDNA) and polymorphic cDNA sequences of the *Dt*DRS family. Branches containing cDNA-derived peptides are indicated by asterisks at nodes; branches with three-disulfide-bridged members are colored *red*, in which *Dl*DRS-10|gDNA is derived from *D. lutescens* TK.

**Figure 6:**
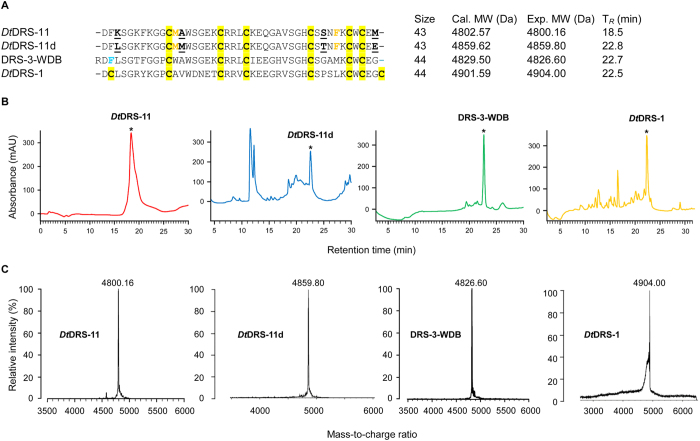
Peptides preparation. **(A)** Sequence alignment. Different amino acids are underlined once and boldfaced; **(B)** RP-HPLC showing retention times (T_*R*_) of the peptides, indicated by asterisks; (**C)** MALDI-TOF MS of HPLC-purified peptides.

**Figure 7:**
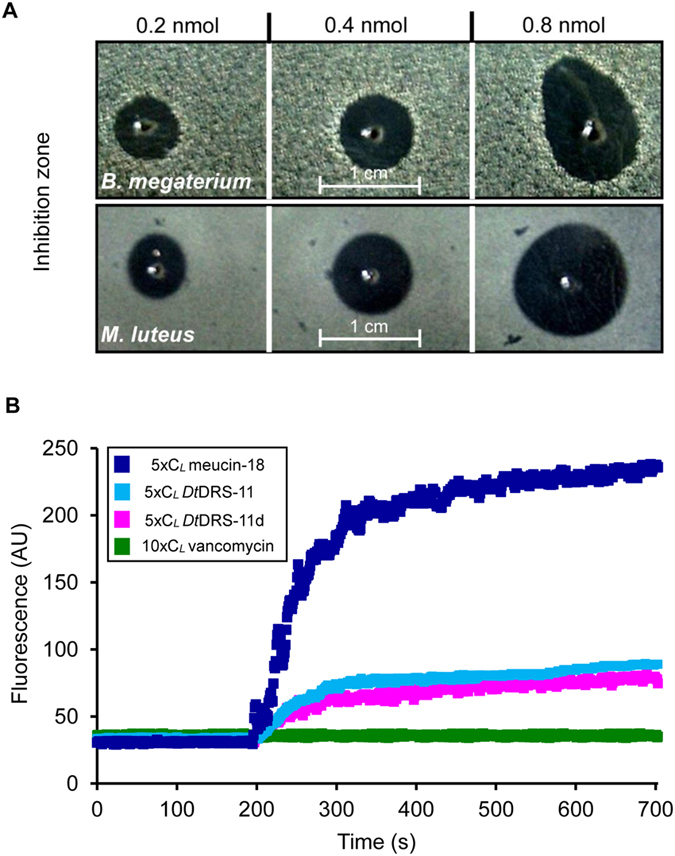
Functional features of WDB-deficient *Dt*DRS peptides. **(A)** Concentration-dependent growth inhibition of *Dt*DRS-11d on *B. megaterium* and *M. luteus.* (**B)** Effect of *Dt*DRS-11 and *Dt*DRS-11d on membrane integrity of *B. megaterium.* Meucin-18 and vancomycin were used as positive and negative controls, respectively^21^,^48^.

**Figure 8:**
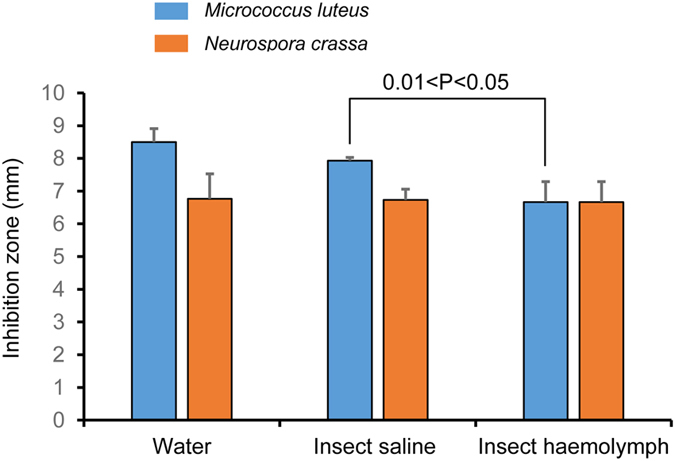
Comparison of antimicrobial activity of *Dt*DRS-11 against *Micrococcus luteus* and *Neurospora crassa* in different environments. The peptide was disolved in water, insect saline^24^ or insect haemolymph extracted from the fifth instar larvae of *Mythimna separate*, respectively, for inhinition zone assay. The haemolymph was centrifuged and its supernatant was used in this assay.

**Figure 9:**
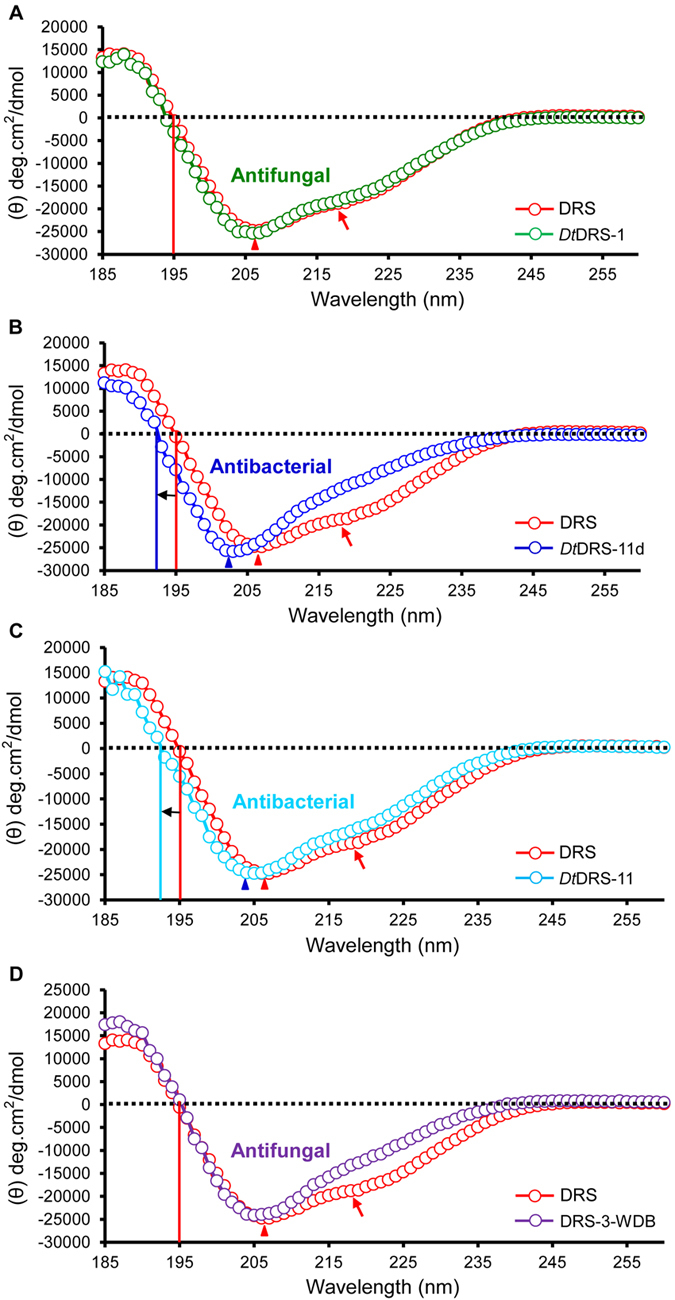
Comparison of CD spectra between DRS and other related peptides. (**A**) *Dt*DRS-1 and DRS; (**B**) *Dt*DRS-11d and DRS; (**C**) *Dt*DRS-11 and DRS; and (**D**) DRS-3-WDB and DRS. The CD results are expressed as mean residue molar ellipticity (θ). Dotted lines show the baseline and black arrows indicate a blue shift in the CD spectra. Red arrows show a unique negative band at 217–218 nm. Red triangle indicates a signature band for the presence of a CSαβ structure, whereas other color triangles indicate alterations as compared to the signature band in DRS.

In addition to the absence of WDB, one might argue that the target’s alteration in the two naturally occurring WDB-deficient variants is also likely associated with other amino acid site mutations. To answer this question, we compared amino acid sequences between the four-disulfide DRSs (antifungal) and the WDB-deficient homologues (antibacterial) (Fig. 3) and found that these homologues only contain two group-specific residues at sites 12 (Met) and 37 (Phe) (numbered according to *Dt*DRS-11) whose location respectively corresponds to the N-terminal loop (n-loop) preceding the α-helix and the γ-core linking two β–strands of DRS (Fig. 1), two regions previously identified as key antibacterial elements of insect defensins^35^,^36^,^41^. These two sites are occupied by hydrophobic side-chains and are situated on one side of the molecule, especially in the functional region of the structurally similar antibacterial insect defensins, providing a structural basis for its antibacterial function. Therefore, if we consider that the rigid structure destruction by the loss of WDB is a prerequisite for the target’s alteration in *Dt*DRS-11 and *Dt*DRS-11d, the two group-specific residues could play a secondary role in further increasing the molecular flexibility following the loss of WDB. This is further strengthened by the structural and functional data of DRS-3-WDB which lacks the two specific residues (Fig. 6). Artificial deletion of WDB in DRS-3 leading to no target’s transfer suggests that the evolutionary emergence of antibacterial function in an ancestral four-disulfide DRS scaffold is a gradual process, in which the WDB loss and mutations in key regions are involved.

It is long accepted that DBs have been added to proteins during evolution to enhance their stability for a fluctuating cellular environment^42^,^43^. DB reshuffling is also found in the evolution of an ape placental ribonuclease^44^. However, the loss of DBs in protein evolution is rarely reported. Herein we show that evolutionary loss of DBs might represent a new mechanism for functional diversification of antifungal peptides. For the *Dt*DRS multigene family, the WDB loss can be considered as an evolutionary advantage for neofunctionalization of duplicated copies in a specific lineage through increasing structural flexibility to alter the target of a member.

## Materials and Methods

### cDNA Cloning

Microbial challenge was performed by pricking of *D. takahasii* adults with a thin needle previously dipped into a concentrated microbial culture of *Micrococcus luteus* (Gram-positive bacterium) and *Neurospora crassa* (filamentous fungus). Total RNA was prepared from either non-challenged or challenged *D. takahasii* adults with Total RNA Isolation Reagent and its reverse transcription to the first-strand cDNA was performed by the EasyScript First-Strand cDNA Synthesis Kit primed by a universal oligo(dT)-containing adaptor primer (dT3AP)^19^. Reverse transcription PCR (RT-PCR) was carried out by a forward primer designed based on the genomic DNA sequence of a predicted *DtDRS* gene (Table S1) combined with the universal reverse primer 3AP^3^. PCR products were ligated into pGM-T vector and resultant recombinant plasmids were transformed into *E. coli* DH5α. Recombinant plasmids were sequenced with T7 and SP6 primers.

### Preparation of Peptides

Linear *Dt*DRS-11 and DRS-3-WDB were chemically synthesized by ChinaPeptides Co., Ltd. (Shanghai, China) and *Dt*DRS-11d by SBS Genetech Co., Ltd (Beijing, China). A dimethyl sulfoxide (DMSO)-based method, previously employed for the synthesis of the three disulfide-bridged *Tityus* kappa toxin and some iberiotoxin analogs^45^, was used to prepare oxidized products of *Dt*DRS-11d in an alkaline environment with some modifications. In brief, crude synthetic peptides were dissolved in 100 μl of 10% DMSO/H_2_O solution (v/v) with a peptide concentration of 2 mM. Following 30 min of incubation at room temperature, 900 μl of 0.1 M Tris-HCl buffer (pH 8.5) was added to give a final peptide concentration of 0.2 mM. The mixture was incubated at 25 °C for 48 h. Peptides were purified to homogeneity by reversed-phase high-pressure liquid chromatography (RP-HPLC) with a C_18_ column (Agilent Zorbax 300SB, 4.6 mm × 150 mm, 5 μm). Elution was carried out with a linear gradient from 0 to 60% acetonitrile in 0.05% (v/v) TFA(v/v) within 40 min at a flow rate of 1 ml/min. For *Dt*DRS-11 and DRS-3-WDB oxidative refolding, peptide samples were dissolved in 0.1 MTris–HCl buffer (pH 8.5) to a final concentration of 0.5 mM and incubated at 25 °C for 48 h. Peptides were purified by RP-HPLC.

For recombinant preparation of *Dt*DRS-1, we chose a mutation strategy to make its expression vector from that of *DRS*^46^ given only one amino acid difference (S29V) between them (Fig. 3). Firstly, we designed two back-to-back primers (DRS-S29V-F and DRS-S29V-R) (Supplementary Table S1) to construct the recombinant plasmid pGEX-6P-1-*Dt*DRS-1 by using pGEX-6P-1-*DRS* as template for inverse PCR^46^. Methods for the expression and purification of *Dt*DRS-1 have been described in our previous paper that reported the work of the first prokaryotic production of DRS^46^. In brief, the pGEX-6P-1-*Dt*DRS-1 plasmid was transformed into *E. coli* BL21(DE3)pLysS host cells and the expression of a fusion protein product (glutathione-S-transferase (GST)-*Dt*DRS-1) was induced by 0.1 mM IPTG at an OD_600_ of 0.6. *E. coli* cells were harvested after induction for 4 hr at 37 °C. Fusion proteins were acquired from the supernatant of *E. coli* cell lysate after sonication, followed by affinity chromatography with glutathione-Sepharose 4B beads (GE Healthcare, USA). After washing by PBS buffer (140 mM NaCl, 2.7 mM KCl, 10 mM Na_2_HPO_4_, 1.8 mM KH_2_PO_4_, pH 7.3), fusion proteins were on-column digested with enterokinase (Sinobio Biotech Co. Ltd, Shanghai, China) at 4 °C overnight. Finally, RP-HPLC was applied to separate *Dt*DRS-1 from GST in the same condition as described above.

Purity and molecular masses of all peptides were determined by matrix-assisted laser desorption ionization time-of-flight mass spectrometry (MALDI-TOF MS) on a Kratos PC Axima CFR plus (Shimadzu Co. LTD, Kyoto, Japan).

### Circular Dichroism Spectroscopy

Circular dichroism (CD) spectra of all peptides described here were recorded on Chirascan™ -plus circular dichroism spectrometer (Applied Photophysics Ltd, United Kingdom) at room temperature from 185 to 260 nm with a quartz cell of 1.0 mm thickness. Spectra were measured at a peptide concentration of about 0.10–0.15 mg/ml in water. Data were collected at 1 nm intervals with a scan rate of 60 nm/min.

Secondary structure elements of peptides were estimated in DICHROWEB, an online server for protein secondary structure analyses from CD spectroscopic data (http://dichroweb.cryst.bbk.ac.uk/html/home.shtml). The mothed used was CDSSTR that implements the variable selection method by performing all possible calulations using a fixed number of proteins from the reference set 6 optimised for 185–240 nm^25^. This method probably produces the most accurate analysis results^47^.

### Antimicrobial Assays

Antimicrobial activity of peptides was assessed by the inhibition zone assay^3^,^48^. Membrane permeability assay was performed according to the method previously reported^48^. Sources of microbial strains used here are listed in Supplementary Table S2.

### Multiple Sequence Alignment and Phylogenetic Tree Construction

DRS and its homologous protein sequences were aligned by ClustalX (http://www.clustal.org/clustal2/) and the aligned sequences were then used to construct a neighbor-joining (NJ) tree on the basis of the Poisson substitution model with pairwise deletion of gaps (MEGA5)^49^. The phylogeny of *Drosophila* RP49 was constructed based on their nucleotide sequences by the NJ method with Tajima-Nei substitution model^49^.

## Additional Information

**Accession Codes**: Nucleotide sequences obtained in this study have been deposited in the GenBank database (http://www.ncbi.nlm.nih.gov/) under accession numbers of KC493088 - KC493103 (Supplementary Table S3).

**How to cite this article**: Gao, B. and Zhu, S. The drosomycin multigene family: three-disulfide variants from *Drosophila takahashii* possess antibacterial activity. *Sci. Rep.* **6**, 32175; doi: 10.1038/srep32175 (2016).

## Supplementary Material

Supplementary Information

## Figures and Tables

**Table 1:** Comparison of antimicrobial activity of three- and four-disulfide DRS-type peptides on different microbial stains.

Microorganisms	C_*L*_ (μM)				
	DRS	*Dt*DRS-1	*Dt*DRS-11	*Dt*DRS-11d	DRS-3-WDB
**Fungi**					
*Aspergillus flavus*	N.A	N.A.	N.A.	N.A.	N.A.
*Aspergillus fumigatus* YJ-407*	N.A.	N.A.	N.A.	N.A.	N.A.
*Aspergillus fumigatus* CEA17**	0.30	0.33	N.D.	N.A.	N.D.
*Aspergillus nidulans* A28	0.45	1.25	N.A.	N.A.	N.A.
*Aspergillus nidulans* RCho15	1.73	2.03	N.D.	N.A.	N.D.
*Aspergillus niger*	1.32	2.63	N.A.	N.A.	N.A.
*Geotrichum candidum* CCTCC AY 93038	1.89	2.50	N.A.	N.A.	4.89
*Neurospora crassa* CGMCC 3.1605	0.1	0.3	8.84	N.A.	3.72
*Candida albicans* JX1195	N.A.	N.A	N.A.	N.A.	N.A.
**Gram-positive bacteria**					
*Bacillus megaterium* CGMCC 1.0459	N.A.	N.A.	4.41	0.98	N.A.
*Micrococcus luteus* CGMCC 1.0290	N.A.	N.A.	3.06	1.08	N.A.
*Staphylococcus aureus* CGMCC 1.89	N.A.	N.A.	N.D.	N.A.	N.D.
*Streptococcus pneumoniae* D39	N.A.	N.A.	N.D.	N.A.	N.D.
**Gram-negative bacteria**					
*Alcaligenes faecalis* CGMCC 1.1837	N.A.	N.A.	N.D.	N.A.	N.D.
*Escherichia coli* ATCC 25922	N.A.	N.A.	N.A.	N.A.	N.A.
*Pseudomonas solanacearum*	N.A.	N.A.	N.D.	N.A.	N.D.
*Xanthomonas oryzae pv.oryzae*	N.D.	N.D.	8.84	N.D.	N.A.

Note: C_*L*_, lethal concentration; N.A.: no activity, indicating that no inhibition zone was observed at 0.8–1.0 nmol peptides each well. N.D.: not determined; ^*^Wild-type strain (CGMCC 0386; China General Microbiological Culture Collection Center); ^**^Mutant (*pyrG*^-^). The gene *pyrG* encodes orotidine 5′-monophosphate decarboxylase.

**Table 2:** Comparison of secondary structure element contents (%) of *Dt*DRS-11d, *Dt*DRS-11 and DRS-3-WDB with DRS.

Peptide	α-Helix	β-Sheet	Turns	Unordered	NRMSD	Major Targets
*Dt*DRS-11	21	16	15	48	0.018	Bacteria
*Dt*DRS-11d	18	16	15	53	0.015	Bacteria
DRS-3-WDB	19	22	18	41	0.017	Fungi
DRS	20	27	19	34	0.025	Fungi

Note: The secondary structure element contents were estimated from the CD data by CDSSTR. NRMSD (normalized root-mean-square deviation) was used to compare how well the best calculated structure correlates with the experimental data^26^.
