# Supplementary material for: The drosomycin multigene family: three-disulfide variants from Drosophila takahashii possess antibacterial activity
Source: Sci Rep. 2016 Aug 26;6:32175. doi: 10.1038/srep32175 (PMC4999892; doi:10.1038/srep32175)
Supplement: Supplementary Information [file srep32175-s1.pdf]

Supplementary information

The drosomycin multigene family: three-disulfide variants from *Drosophila takahashii* possess antibacterial activity

Bin Gao, Shunyi Zhu

Supplementary Table S1 PCR primers used in this study

**Name**	**Sequence**
*Dt*DRS-1-F	5'-ATGATGCAGATGAAGTACTTG-3'
*Dt*DRS-2/4/9-10-F	5'-ATGRTGCAGATCAARTTCCTGT-3'
*Dt*DRS-2F	5'-AGGCCGACTGTCTTTCGGGC-3'
*Dt*DRS-4F	5'-TCCTGTTTGCCCTGGCTGTT-3'
*Dt*DRS-3/5-F	5'-GCTGTCCTGATGCTGATCGT-3'
*Dt*DRS-6-F	5'-ATGTCGCTTAACAAAAGCCTG-3'
*Dt*DRS-7/8-F	5'-ATCAAGTACTTGYTCGCCCTC-3'
*Dt*DRS-11-F	5'-TCCTGGCCGTGATGACAATTG-3'
*Dl*DRS-11-F	5'-GTCGACTTATTCTTCGCACCAACAT-3'
*Dl*DRS-11-R	5'-TTATTCTTCGCACCAACAT-3'
Rp49-F	5'-AAGATCGTGAAGAAGCGCACCA-3'
DRS-S29V-F	5'-GTCAGTGGCCACTGCAGCCCCAGT-3'
DRS-S29V-R	5'-GCGTCCCTCCTCCTTGCACACACG-3'

Note: R=A/G; Y=T/C.

Supplementary Table S2 Sources of microbial strains used in this study

Microorganism	Source
**Fungi**	
Aspergillus flavus	Prof. Yijian Yao, Institute of Microbiology, Beijing, China
Aspergillus fumigatus CEA17	Prof. Cheng Jin, Institute of Microbiology, Beijing, China
Aspergillus fumigatus YJ-407	Prof. Cheng Jin, Institute of Microbiology, Beijing, China
Aspergillus nidulans A28	Prof. Shaojie Li, Institute of Microbiology, Beijing, China
Aspergillus nidulans RCho15	Prof. Jae-Hyuk Yu, University of Wisconsin, Madison, WI 53706, USA
Aspergillus niger	Prof. Yijian Yao, Institute of Microbiology, Beijing, China
Geotrichum candidum CCTCC AY 93038	China Center for Type Culture Collection, Wuhan University, Wuhan, China
Neurospora crassa CGMCC 3.1605	Center for Microbial Resources, Institute of Microbiology, Beijing, China
Candida albicans JX1195	Prof. Guanghua Huang, Institute of Microbiology, Beijing, China
**Gram-positive bacteria**	
Bacillus megaterium CGMCC 1.0459	Center for Microbial Resources, Institute of Microbiology, Beijing, China
Micrococcus luteus CGMCC 1.0290	Center for Microbial Resources, Institute of Microbiology, Beijing, China
Staphylococcus aureus CGMCC 1.89	Center for Microbial Resources, Institute of Microbiology, Beijing, China
Streptococcus pneumoniae D39	Prof. Jingren Zhang, Tsinghua University, Beijing, China
**Gram-negative bacteria**	
Alcaligenes faecalis CGMCC 1.1837	Center for Microbial Resources, Institute of Microbiology, Beijing, China
Escherichia coli ATCC 25922	Center for Microbial Resources, Institute of Microbiology, Beijing, China
Pseudomonas solanacearum	Prof. Fengming Song, Zhejiang University, Hanzhou, China
Xanthomonas oryzae pv.oryzae	Prof. Fengming Song, Zhejiang University, Hanzhou, China

Supplementary Table S3 The GenBank accession numbers of genes (cDNAs) isolated in this study

**Gene name**	**Accession number**
*Dt*DRS-1	KC493088
*Dt*DRS-1a	KC493089
*Dt*DRS-1b	KC493090
*Dt*DRS-2-1	KC493091
*Dt*DRS-4	KC493095
*Dt*DRS-4a	KC493096
*Dt*DRS-6a	KC493092
*Dt*DRS-6b	KC493093
*Dt*DRS-6c	KC493094
*Dt*DRS-11d	KC493097
*Dt*DRS-11c	KC493098
*Dt*DRS-11b	KC493099
*Dt*DRS-11a	KC493100
*Dt*DRS-11-1	KC493101
*Dt*DRS-11-2	KC493102
*Dt*DRS-11-3	KC493103

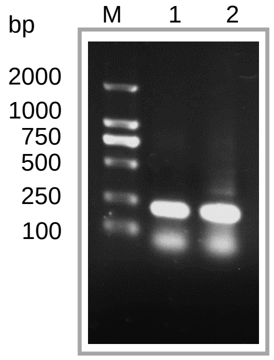

Figure S1. Genomic PCR of three-disulfide DRS-type peptides from *D. takahashii* IR (lane 1) and *D. lutescens* TK (lane 2)***.***

```
DtDRS-1|gDNA          -DCLSGRYKGPCAVWDNETCRRVCKEE-GRVSGHCSPSLKCWCEGC
DtDRS-1|cDNA*         -DCLSGRYKGPCAVWDNETCRRVCKEE-GRVSGHCSPSLKCWCEGC
DtDRS-1a|cDNA*        -DCLSGRYKGPCAVWDNETCRRVCKEE-GRVSGHCSPSLKCWCEGC
DtDRS-1b|cDNA*        -DCLSGRYKGPCAVWDNETCRRVCKEE-GRVSGHCSPSLKCWCEGC
DtDRS-2|gDNA          -DCLSGRYGGPCAVWDNETCRRVCKEE-GRTSGHCSPSLKCWCEGC
DtDRS-2-1|cDNA*       -DCLSGRYGGPCAVWDNETCRRVCKEE-GRSSGHCSPSLKCWCEGC
DtDRS-3|gDNA          VDCLSGRYRGGCYVWSKEKCKRFCISE-GRSGGHCSPSLKCWCEGC
DtDRS-4|gDNA          SDCMSGKFTGPCFAWDGELCRRLCKEE-GRVSGHCSAGLACWCEEC
DtDRS-4|cDNA*         SDCMSGKFTGPCFAWDGELCRRLCKEE-GRVSGHCSAGLACWCEEC
DtDRS-4a|cDNA*        SDCMSGKFTGPCFAWDGELCRRLCKEE-GRVSGHCSAGLACWCEEC
DtDRS-5|gDNA          VDCLSGRFRGSCPVWSNKKCKNTCIRE-GRRSGHCSPSLKCWCEGC
DtDRS-6|gDNA          YDCLSGKFSGPCWAWDGEQCRRLCTEE-GHVSGHCSASLKCWCEGC
DtDRS-6a|cDNA*        YDCLSGKFSGPCWAWDGEQCRRLCIEE-GHVSGHCSASLKCWCEGC
DtDRS-6b|cDNA*        YDCLSGKFSGPCWAWDGEQCRRLCIEE-GHVSGHCSASLKCWCEGC
DtDRS-6c|cDNA*        YDCLSGKFSGPCWAWDGEQCRRLCIEE-GHVSGHCSASLKCWCEGC
DtDRS-7|gDNA          -DCLSGRYRGPCAIWDNETCRRICREE-RRVSGHCSARLQCWCEGC
DtDRS-8|gDNA          -DCLSGRFRGPCAVWDWERCRRICREEERRLSGHCSARLQCWCEGC
PseudoDtDRS|gDNA      FDCMSGMYSGPCFSWNTEHCRRLCKEE-GRVSGHCSPRLACWCEDC
DtDRS-9|gDNA          -DFLSGKFKGGCMAWSREKCRRLCKEE-GGVSGHCTT-FKCWCEQ-
DtDRS-10|gDNA         -DFLSGKFKGGCMMWSTEKCRRLCKEE-GGVSGHCSTNFKCWCEQ-
DtDRS-11|gDNA         -DFKSGKFKGGCMAWSGEKCRRLCKEQ-GAVSGHCSSNFKCWCEM-
DtDRS-11a|cDNA*       -DFLSGKFKGGCMMWSGEKCRRLCKEQ-GAVSGHCSTNFKCWCEE-
DtDRS-11b|cDNA*       -DFLSGKFKGGCMMWSGEKCRRLCKEQ-GAVSGHCSTNFKCWCEE-
DtDRS-11c|cDNA*       -DFLSGKFKGGCMMWSGEKCRRLCKEQ-GAVSGHCSTNFKCWCEE-
DtDRS-11d|cDNA*       -DFLSGKFKGGCMMWSGEKCRRLCKEQ-GAVSGHCSTNFKCWCEE-
DtDRS-11-1|cDNA*      -DFLSGKFKGGCMMWSGEKCRHLCKEQ-GAVSGHCSTNFKCWCEE-
DtDRS-11-2|cDNA*      -DFLSGKFKGGCMMWSTEKCRRLCKEQ-GAVSGHCSTNFKCWCEE-
DtDRS-11-3|cDNA*      -DFLSGKFKGGCMMWSGEECRRLCKEQ-GAVSGHRSTNFKCWCEE-
DlDRS-10|gDNA         -DFLSGKFKGGCMAWSGEKCRRLCKEE-GGVSGHCSSNFKCWCEE-
```

Figure S2. Comparison of amino acid sequences of *Dt*DRSs deduced from cDNA (indicated by "*") clones and genomic DNA of *D. takahasii* IR. *Dl*DRS-10, a genomic clone derived from *D. lutescens* TK, is also aligned here. Cysteines involved in the formation of disulfide bridges are shadowed in *yellow*. Polymorphic sites are highlighted in *red*.
